# Supplementary material for: HBV prevalence in Sub-continental countries: A systematic review and meta-analysis
Source: PLoS One. 2023 Dec 8;18(12):e0295670. doi: 10.1371/journal.pone.0295670 (PMC10707566; doi:10.1371/journal.pone.0295670)
Supplement: S1 Table — (DOCX) [file pone.0295670.s002.docx]

**SUPPLEMENTARY TABLES**

**Supplementary table 1. Table 1. Summary of the terms used for the systematic search of electronic databases.**

|  | **Search Terms** |
| --- | --- |
| **Population**: People living countries within the Indian subcontinent | Subcontinent OR India OR Pakistan OR Nepal OR Bangladesh OR Sri Lanka OR Bhutan NOT China  **MeSH terms:** N/A |
| **Intervention:** Vaccination or no intervention | Vaccination* OR vaccin* OR no intervention OR immuni?ation  **MeSH terms:** Vaccination |
| **Comparison:** Rest of the world HBV prevalence | Prevalence OR trend AND Worldwide OR global OR world  **MeSH terms:** Prevalence |
| **Outcome:** Prevalence of HBV | HBV OR Hepatitis B* OR chronic HBV OR Hepatitis B infection OR HBV infection  **MeSH terms:** Hepatitis B virus, Hepatitis B, Hepatitis B Epidemiology |
