# Supplementary material for: HBV prevalence in Sub-continental countries: A systematic review and meta-analysis
Source: PLoS One. 2023 Dec 8;18(12):e0295670. doi: 10.1371/journal.pone.0295670 (PMC10707566; doi:10.1371/journal.pone.0295670)
Supplement: S1 Fig — The shape of this plot indicates the large amount of variation between studies included in this review, and indicates the possibility that some publication bias exists. (DOCX) [file pone.0295670.s004.docx]

**SUPPLEMENTARY FIGURE**


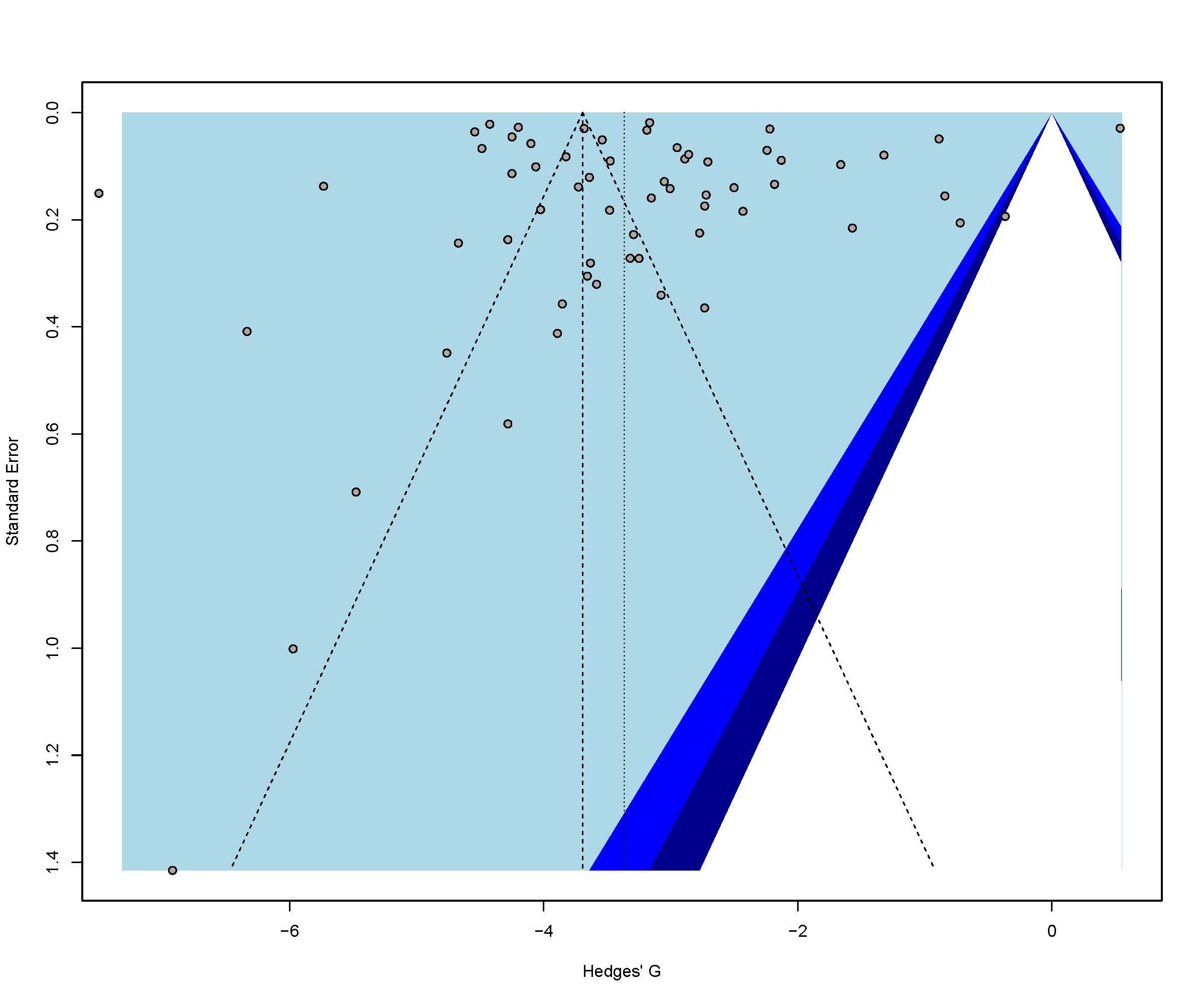


**Supplementary Figure 1.** Funnel plot showing the chance of publication bias having an effect on study results. The shape of this plot indicates the large amount of variation between studies included in this review, and indicates the possibility that some publication bias exists.
