# Supplementary material for: HBV prevalence in Sub-continental countries: A systematic review and meta-analysis
Source: PLoS One. 2023 Dec 8;18(12):e0295670. doi: 10.1371/journal.pone.0295670 (PMC10707566; doi:10.1371/journal.pone.0295670)
Supplement: Attachment — Submitted filename: Article review_HBV prevalence (2).docx [file pone.0295670.s005.docx]

**HBV prevalence in Sub-continental countries: a systematic review and meta-analysis**

**Review:**

Hepatitis B is a global public health threat and the world’s most common serious liver infection. It is up to 100 times more infectious than the HIV/AIDS virus. According to Hepatitis B Foundation (USA) 2 billion people have been infected with the virus (1 out of 3 people). Approximately 1.5 million people become newly infected each year. Almost 300 million people are chronically infected.

In this systematic review and meta-analysis, the authors sought to ascertain the prevalence of HBV infection among populations within the subcontinental countries, namely India, Pakistan, Bangladesh, Nepal, Sri Lanka and Bhutan. The authors did not specify the study design was for inclusion, however due to the nature of the outcome of interest, most studies were cross-sectional. A secondary aim of the study was also to assess the vaccination coverage within the study populations.

There are some of the points that needed to be addressed:

1. Page number 7, Line number 159-177 (Data synthesis and analysis)

- The use of random effects models is justified, but the reason for selecting these models could be further elaborated. Also, the statement "variance between identified studies was due to more than only selection bias or sampling errors" needs clarification as it is unclear what other sources of variance are considered.
- The I^2^ statistic is mentioned without any interpretation of what it means. The reader should be informed about the level of heterogeneity and its implications for the meta-analysis results.
- There is a mention of the software packages used for data analysis, but it doesn't clarify why two different packages were used. It should be explained why specific packages were chosen and if there was any specific reason to use Stata for visualizing figures.

2. Page number 18, Line number 264: The sentence is incomplete (Remains…)
3. Page number 19, line number 292: please correct the sentence (“at-risk” groups…)
4. Page number 19, line number 298: please correct the sentence (“at-risk” behaviors…)
5. Page number 20, line number 303: please correct the sentence (Focused and focusing used in the same sentence)
6. Page number 20, line number 304, 305, 306, 308, 309, 310: please correct the sentence (the word “population” is repeated 2 and 3 times)
7. Page number 22, line number 337, 338: please correct the sentence, it does not have any sense.
