# Supplementary material for: HBV prevalence in Sub-continental countries: A systematic review and meta-analysis
Source: PLoS One. 2023 Dec 8;18(12):e0295670. doi: 10.1371/journal.pone.0295670 (PMC10707566; doi:10.1371/journal.pone.0295670)
Supplement: Attachment — Submitted filename: Plos One Response to Reviewers 2.docx [file pone.0295670.s006.docx]

Dear editors,

Thank you for your time and advice for adjustments needed within our manuscript. Please see changes below as per your recommendations.

*“1. We notice that your supplementary figures are uploaded with the file type 'Figure'. Please amend the file type to 'Supporting Information'. Please ensure that each Supporting Information file has a legend listed in the manuscript after the references list.”*

These changes have now been implemented in both of the new “Revised Manuscript” files which have been uploaded and approved by the corresponding author.

We thank you again for the time spent reviewing the submission and hope that it is now up to date with the required guidelines.
